# Public Electric Baths in Japan: A Risk of Inappropriate Implantable Cardioverter‐Defibrillator Shock

**DOI:** 10.1002/joa3.70298

**Published:** 2026-02-17

**Authors:** Takahisa Ido, Takashi Nakashima, Makoto Yamaura, Shigekiyo Takahashi, Takuma Aoyama

**Affiliations:** ^1^ Department of Cardiology Central Japan International Medical Center Gifu Japan; ^2^ Department of Cardiology, Graduate School of Medicine Gifu University Gifu Japan; ^3^ Department of Molecular Pathophysiology Shinshu University Graduate School of Medicine Nagano Japan

**Keywords:** electric bath, ICD, shock, ventricular fibrillation

## Abstract

This case represents the first reported instance of an inappropriate S‐ICD shock caused by a public electric bath in Japan.

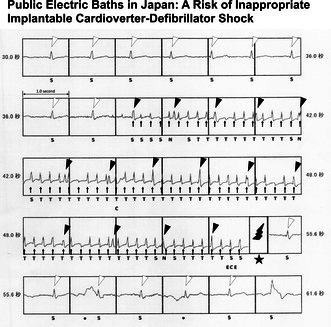

## 1 Case Presentation

A 50‐year‐old man was diagnosed with idiopathic ventricular fibrillation (VF) after experiencing a cardiopulmonary arrest due to VF three years earlier. A subcutaneous implantable cardioverter‐defibrillator (S‐ICD; EMBLEM MRI S‐ICD, model A219, Boston Scientific, Natick, MA) was implanted for secondary prevention. The S‐ICD sensing filter (SMART Pass) was activated. The sensing vector was set to the secondary vector, which is recorded between the distal ring on the lead and the can. The shock zone was programmed at 240 beats per minute (cycle length: 250 milliseconds [ms]). The patient was instructed to avoid “denki‐buro” (electric baths), a common feature in Japanese public baths where a mild electric current is discharged into the water.

**FIGURE 1:**
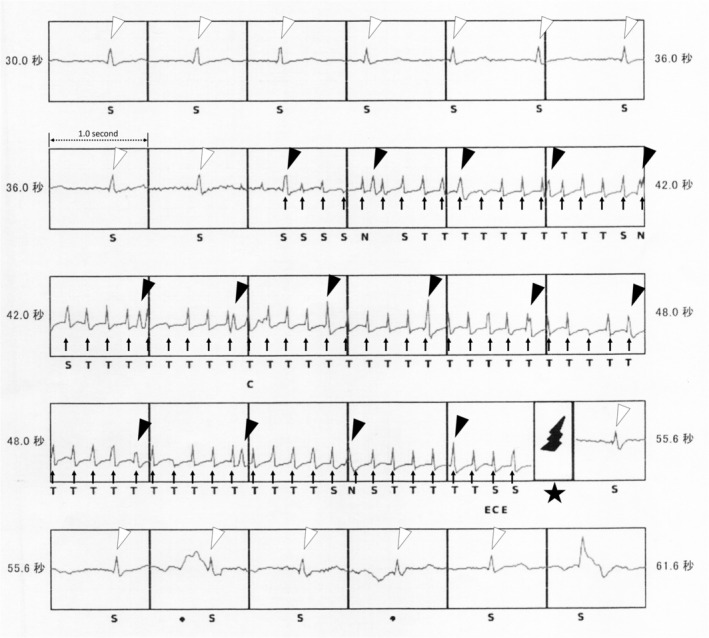
The tracing shows an inappropriate S‐ICD shock induced by the “denki‐buro.” High‐frequency signals with a cycle length of approximately 200 ms are detected as ventricular fibrillation (arrows), resulting in shock delivery (star). Dissociated sinus QRS complexes are also present (black and white arrowheads).

During a routine outpatient follow‐up, device interrogation revealed an ICD shock (Figure 1). The pacing threshold, sensing sensitivity, and lead impedance were all within normal limits. A medical interview revealed that the patient had fallen in a public bathhouse and had accidentally immersed his left upper limb—from the fingertips to the shoulder—in the “denki‐buro” at the time the S‐ICD shock was delivered. No other body parts were submerged. He had no predisposing symptoms. Simultaneously with the defibrillator discharge, he withdrew his left upper limb from the water.

The intracardiac electrogram revealed high‐frequency signals with a cycle length of approximately 200 ms. These signals were interpreted as VF and subsequently triggered shock delivery (arrows and star in Figure 1). During VF detection, regular signals with a cycle length of 1000–1100 ms were also present (black arrowheads). These signals were dissociated from the high‐frequency signals, showed relatively regular intervals, and were similar to those recorded before VF detection (white arrowheads). Therefore, the regular signals observed during VF detection were presumed to represent QRS complexes of the underlying sinus rhythm. The high‐frequency signals interpreted as VF were, in fact, external noise generated by the “denki‐buro.” Based on the medical interview and the intracardiac electrogram findings, we concluded that the ICD shock had been inappropriately delivered due to electric currents from the “denki‐buro.”

Inappropriate ICD shocks may be caused by external electromagnetic interference and have been associated with increased mortality [1]. A “denki buro,” is relatively common in Japan [2]. In Japan, the use of “denki‐buros” is prohibited for patients with cardiac implantable electronic devices. However, inappropriate ICD shocks induced by “denki‐buros” have rarely been reported. Inappropriate ICD shocks induced by a public “denki‐buro” have previously been reported in a patient with a transvenous ICD [2]. To the best of our knowledge, no such cases have been reported in patients with an S‐ICD.

This is the first reported case of an inappropriate ICD shock caused by a “denki‐buro” in a patient with an S‐ICD. Physicians should be aware that a “denki‐buro” may trigger inappropriate ICD shocks in patients with ICDs. Inappropriate shocks can occur despite prior patient awareness. Patients, as well as public bath operators, should be informed of this potential risk.

## Funding

The authors have nothing to report.

## Ethics Statement

This study was approved by the institutional review board of Central Japan International Medical Center.

## Consent

Informed consent was obtained from the patient.

## Conflicts of Interest

The authors declare no conflicts of interest.

## Data Availability

The data that support the findings of this study are available from the corresponding author upon reasonable request.
